# Short telomere length in autoimmune neutropenia of childhood: A mere coincidence or an association?

**DOI:** 10.1002/jha2.689

**Published:** 2023-05-10

**Authors:** Hamza S. Gorsi, Maria Gallardo‐Rios, Süreyya Savaşan

**Affiliations:** ^1^ Division of Hematology/Oncology Children's Hospital of Michigan Detroit Michigan USA; ^2^ Department of Pediatrics Central Michigan University Mt Pleasant Michigan USA; ^3^ Children's Hospital of Michigan Bone Marrow Transplant Program Barbara Ann Karmanos Cancer Center Detroit Michigan USA

**Keywords:** autoimmune neutropenia of childhood, short telomere length

1

Telomeres protect chromosomes from degradation and fusion [1, 2]. They shorten with successive cell divisions leading to eventual senescence [3]. Short telomere length has been found in several disorders, including dyskeratosis congenita (DKC) and idiopathic pulmonary fibrosis. Presentation time of short telomere syndromes ranges from early childhood to adulthood with some evidence of genetic anticipation [4]. Dyskeratosis congenita, a bone marrow failure syndrome characterized by short telomeres, has never been associated with autoimmune neutropenia (AIN). Here, we report our experience on coexisting AIN and short telomeres in two children.

Case 1. A female patient presented with severe neutropenia (0.1 × 10^9^/L) at 1 year of age without history of frequent or serious infections. Family history was negative for neutropenia. She tested positive for anti‐neutrophil antibodies at different times consistent with AIN. There are no dysplastic nails or persistent oral leukoplakia. As part of initial work‐up, telomere length analysis was performed and showed short telomeres (<1%) in both granulocytes and lymphocytes. Her parents do not have short telomeres. A variant of unknown significance (VUS) in DKC‐related *DKC1* was detected in a gene panel for inborn errors of immunity and later, a VUS in *ACD* detected in whole exome sequencing (WES). Bone marrow biopsy showed 90% cellularity and trilineage hematopoiesis with prominent neutrophil hemophagocytosis.

Case 2. A female patient with positive antineutrophil antibodies and so far, a benign course without infections presented with neutrophils of 0.3 × 10^9^/L at 3 years of age. She has a patchy hypo/hyper‐pigmented skin rash on right anterior chest not crossing the midline without any dysplastic nails. Due to recurrent oral ulcer history, telomere length analysis was performed and showed short telomeres (<1%) in both granulocytes and lymphocytes. A pathological heterozygous *RTEL1* mutation was identified on WES. Her father was found to carry the same mutation and does not have any health problems at age 42.

While normal neutrophil expression of CD11b, CD11c, CD16, and CD18 were observed, CD11a‐negative neutrophil populations were observed by flow cytometric analysis in both children (Table 1). Presence of bone marrow neutrophil phagocytosis and CD11a‐negative neutrophils may be indirect reflections of ongoing antibody‐mediated immune reaction.

**TABLE 1:** Patient clinical and laboratory characteristics.

Case	Current age	Gender	Mouth sores	ANC	Neutrophil Ab	CD11a	TL‐G	TL‐L	Genes
1	43 months	Female	1 episode	0.1–1.2	Positive x 2	47.9%	<1%	<1**%**	ACD c.1262G>A [VUS] DKC c.294‐9C>T [VUS]
2	36 months	Female	3 episodes	0.3–3.6	Positive x 2	28.9%	<1%	<1%	RTEL1 c.2956C>T [P} ABCG8 c.55G>G (HRA] RIPK1 c.977T>A [VUS]

Abbreviations: Ab, antibodies; ANC, absolute neutrophil count, reflecting the range during the follow up (×10^9^/L); CD11a reflects peripheral blood neutrophil percent with negative surface CD11a expression, healthy controls have 100 percent positive expression; HRA, high risk allele. P, pathogenic; TL‐G, telomere length in granulocytes; TL‐L, telomere length in lymphocytes; VUS, variant of uncertain significance.

Clinical course has been consistent with AIN of childhood in both patients with positive antineutrophil antibodies and lack of infections [5]. The contribution of short telomeres to the clinic picture currently remains unclear. Furthermore, the etiology of short telomere length is also puzzling, although two different VUS have been observed in DKC‐related genes in the first case. The *RTEL1* mutation reported in second patient is known to be associated with broad spectrum short telomere syndromes. Although both conditions are rare, comparatively, childhood AIN is by far more common than short telomere syndromes. High cell turnover secondary to immune destruction of neutrophils in AIN cannot account for short telomeres in lymphocytes in our patients.

Whether the association between AIN and short telomeres is coincidental or points at a mechanistic relationship remains unknown. And if short telomeres constitute a biological environment facilitating antineutrophil antibody development warrants further research. Nevertheless, a recent report of positive neutrophil antibody test in 5 of 14 of *ELANE*‐mutated severe congenital neutropenia or cyclical neutropenia cases reaching 36% incidence extends the discussion of autoimmune response against genetically modified neutrophils, although the hypothetical mechanism can be different in our cases [6]. These two cases reported here furthermore, point to keeping an open mind about potential extension of investigations in selected cases of AIN.

## AUTHOR CONTRIBUTIONS

Drafted and edited the manuscript: HSG. Edited the manuscript and handled the regulatory process: MG‐R. Planned the report and edited the manuscript: SS.

## CONFLICT OF INTEREST STATEMENT

The authors declare that there is no conflict of interest that could be perceived as prejudicing the impartiality of the research reported.

## FUNDING INFORMATION

The authors received no specific funding for this work.

## ETHICS STATEMENT

Went through IRB questionnaire and it was determined: The Institutional Review Board Leadership has determined this project does not meet the definition of human subject research under the purview of the IRB according to federal regulations. Verbal consent was obtained from families.

## Data Availability

Data sharing is not applicable to this article as no new data were created or analyzed in this study.
